# A Type IV Pilus Mediates DNA Binding during Natural Transformation in *Streptococcus pneumoniae*

**DOI:** 10.1371/journal.ppat.1003473

**Published:** 2013-06-27

**Authors:** Raphaël Laurenceau, Gérard Péhau-Arnaudet, Sonia Baconnais, Joseph Gault, Christian Malosse, Annick Dujeancourt, Nathalie Campo, Julia Chamot-Rooke, Eric Le Cam, Jean-Pierre Claverys, Rémi Fronzes

**Affiliations:** 1 Institut Pasteur, Groupe Biologie Structurale de la Sécrétion Bactérienne, Paris, France; 2 CNRS, UMR3528, Paris, France; 3 Maintenance des Génomes et Microscopies Moléculaire, UMR 8126 CNRS-Université Paris Sud, Institut Gustave Roussy, Villejuif, France; 4 Institut Pasteur, Unité de Spectrométrie de Masse Structurale et Protéomique, Paris, France; 5 CNRS, UMR5100, Toulouse, France; 6 Université de Toulouse, UPS, Laboratoire de Microbiologie et Génétique Moléculaires, Toulouse, France; The University of Texas Health Science Center at San Antonio, United States of America

## Abstract

Natural genetic transformation is widely distributed in bacteria and generally occurs during a genetically programmed differentiated state called competence. This process promotes genome plasticity and adaptability in Gram-negative and Gram-positive bacteria. Transformation requires the binding and internalization of exogenous DNA, the mechanisms of which are unclear. Here, we report the discovery of a transformation pilus at the surface of competent *Streptococcus pneumoniae* cells. This Type IV-like pilus, which is primarily composed of the ComGC pilin, is required for transformation. We provide evidence that it directly binds DNA and propose that the transformation pilus is the primary DNA receptor on the bacterial cell during transformation in *S. pneumoniae*. Being a central component of the transformation apparatus, the transformation pilus enables *S. pneumoniae*, a major Gram-positive human pathogen, to acquire resistance to antibiotics and to escape vaccines through the binding and incorporation of new genetic material.

## Author Summary

Natural genetic transformation, first discovered in *Streptococcus pneumoniae* by Griffith in 1928, is observed in many Gram-negative and Gram-positive bacteria. This process promotes genome plasticity and adaptability. In particular, it enables many human pathogens such as *Streptococcus pneumoniae, Staphylococcus aureus* or *Neisseria gonorrhoeae* to acquire resistance to antibiotics and/or to escape vaccines through the binding and incorporation of new genetic material. While it is well established that this process requires the binding and internalization of external DNA, the molecular details of these steps are unknown. In this study, we discovered a new appendage at the surface of *S. pneumoniae* cells. We show that this appendage is similar in morphology and composition to appendages called Type IV pili commonly found in Gram-negative bacteria. We demonstrate that this new pneumococcal pilus is essential for transformation and that it directly binds DNA. We propose that the transformation pilus is an essential piece of the transformation apparatus by capturing exogenous DNA at the bacterial cell surface.

## Introduction

Natural transformation, first discovered in *Streptococcus pneumoniae* [1], is observed in many Gram-negative and Gram-positive bacteria [2]. It increases bacterial adaptability by promoting genome plasticity through intra- and inter-species genetic exchange [3]. In *S. pneumoniae*, a major human pathogen responsible for severe diseases such as pneumonia, meningitis and septicemia, transformation is presumably responsible for capsular serotype switching and could therefore reduce the efficiency of capsule-based vaccines after a short period [4]. In this species, it occurs during a genetically programmed and differentiated state called competence that is briefly induced at the beginning of exponential growth. During this competent state, pneumococci secrete a peptide pheromone called Competence-Stimulating-Peptide (CSP) [5], which spreads competence in the pneumococcal population. Interestingly, in *S. pneumoniae*, some antibiotics and DNA-damaging agents induce competence, which would act as an alternative SOS response and ultimately increases bacterial resistance to external stresses [6].

During transformation, environmental DNA is bound at the surface of competent cells and transported through the cell envelope to the cytosolic compartment. This process has been mostly studied in the Gram-positive bacterium *Bacillus subtilis* with additional information coming from studies in *S. pneumoniae* [7], [8]. In both species, a DNA translocation apparatus mediates the transfer of DNA through the cellular membrane. In *S. pneumoniae*, it is composed of ComEA, EndA, ComEC and ComFA. Incoming double-stranded DNA would bind the membrane receptor ComEA. One DNA strand crosses the membrane through ComEC while the endonuclease EndA degrades the other strand. On the cytoplasmic side, ComFA, an ATPase that contains a helicase-like domain, would facilitate DNA internalization through ComEC. Once inside the bacterium, single-stranded DNA is either integrated into the chromosome by RecA-mediated homologous recombination or entirely degraded.

**Figure 1:**
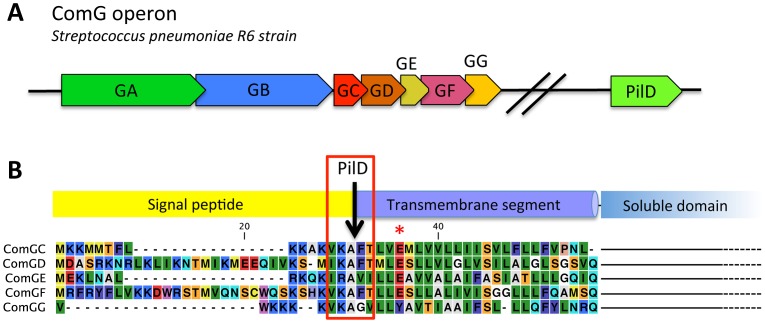
Genes potentially essential for transformation pilus assembly in *S. pneumonia and* prepilin sequences. (**A**) Genes that are potentially essential in transformation pilus assembly. The *comG* operon contains 7 genes named *comGA* to *comGG*. A gene that encodes a pre-pilin peptidase homolog, *pilD*, is found elsewhere on the chromosome. The name used to designate the *comG* and *pilD* genes varies in different pneumoccocal strains. For clarity, we refer to the comG nomenclature used in B. subtilis. Names of the corresponding genes in different S. pneumoniae strains are found in the Table S1. (**B**) Alignment of the N-terminal extremity of the five possible prepilins show that ComGC, ComGD, ComGE and comGF contain a canonical prepilin cleavage motif (red rectangle) [20]. All four proteins also contain a conserved glutamic acid residue in position 5 after the cleavage site (red asterisk), a conserved feature of all Type IV pilins [43]. ComGG has a degenerated peptidase motif and does not contain a conserved glutamic acid in position 5. Secondary structure predictions for the five proteins propose a N-terminal hydrophobic helix and a C-terminal soluble domain of variable size (not shown in the figure), the typical topology found in Type IV pilins.

Strikingly, all transformable Gram-positive bacteria also carry a *comG* operon that resembles operons encoding Type IV pili and Type II secretion pseudopili in Gram-negative bacteria, as well as a gene encoding a prepilin peptidase homolog, *pilD* [7]. In *B. subtilis* and *S. pneumoniae*, *comG* and *pilD* genes are exclusively expressed in competent cells and are essential for transformation [9], [10], [11]. In *S. pneumoniae*, the *comG* operon encodes a putative ATPase (ComGA), a polytopic membrane protein (ComGB) and five prepilin candidates named ComGC, ComGD, ComGE, ComGF and ComGG (Figure 1A and B and table S1). By homology with Type IV pili, it is generally proposed that these proteins could be involved in the assembly of a transformation pseudo-pilus at the surface of competent cells [7], [8], [12]. So far, two studies show that a large macromolecular complex containing ComGC can be found at the surface of competent *B. subtilis* cells [9], [12]. In this complex, ComGC subunits appear to be linked together by disulfide bridges [9]. All the other ComG proteins and the PilD homolog, ComC, are necessary for the formation of this complex [9]. It was proposed that this complex could correspond to the transformation pseudo-pilus.

Despite these first clues, no transformation appendage could be directly visualized at the surface of any competent Gram-positive bacterium. In addition, the function of the ComG proteins during transformation remains unclear. Mutations in the cytosolic ComGA protein abolish DNA binding at the surface of both *B. subtilis* and *S. pneumoniae* [13], [14], [15]. This strongly suggests that the ComGC-containing macromolecular complex detected at the surface of competent *B. subtilis* cells could bind DNA. However, it was recently shown that ComGA is the only ComG protein essential to the initial DNA binding at the surface of competent *B. subtilis* cells [14]. This protein would interact with an unknown DNA receptor at the surface of competent cells while the other ComG proteins would only be required at a later stage during transformation.

In this study, we provide the first direct evidence for the existence of a transformation pilus in a Gram-positive bacterium. We discovered a new appendage at the surface of competent pneumococci that we could visualize using immuno-fluorescence and electron microscopy. Competent cells harbor one or a few appendages that are morphologically similar to Type IV pili found in Gram-negative bacteria. We were able to purify this pilus and showed that it is essentially composed of the ComGC pilin. We also demonstrate that pilus assembly is required for transformation. As we provide direct evidence that the transformation pilus binds extracellular DNA, we propose it is the primary DNA receptor at the surface of competent pneumococci.

## Results

### An extracellular appendage containing ComGC is detected at the surface of competent *S. pneumoniae*

**Figure 2:**
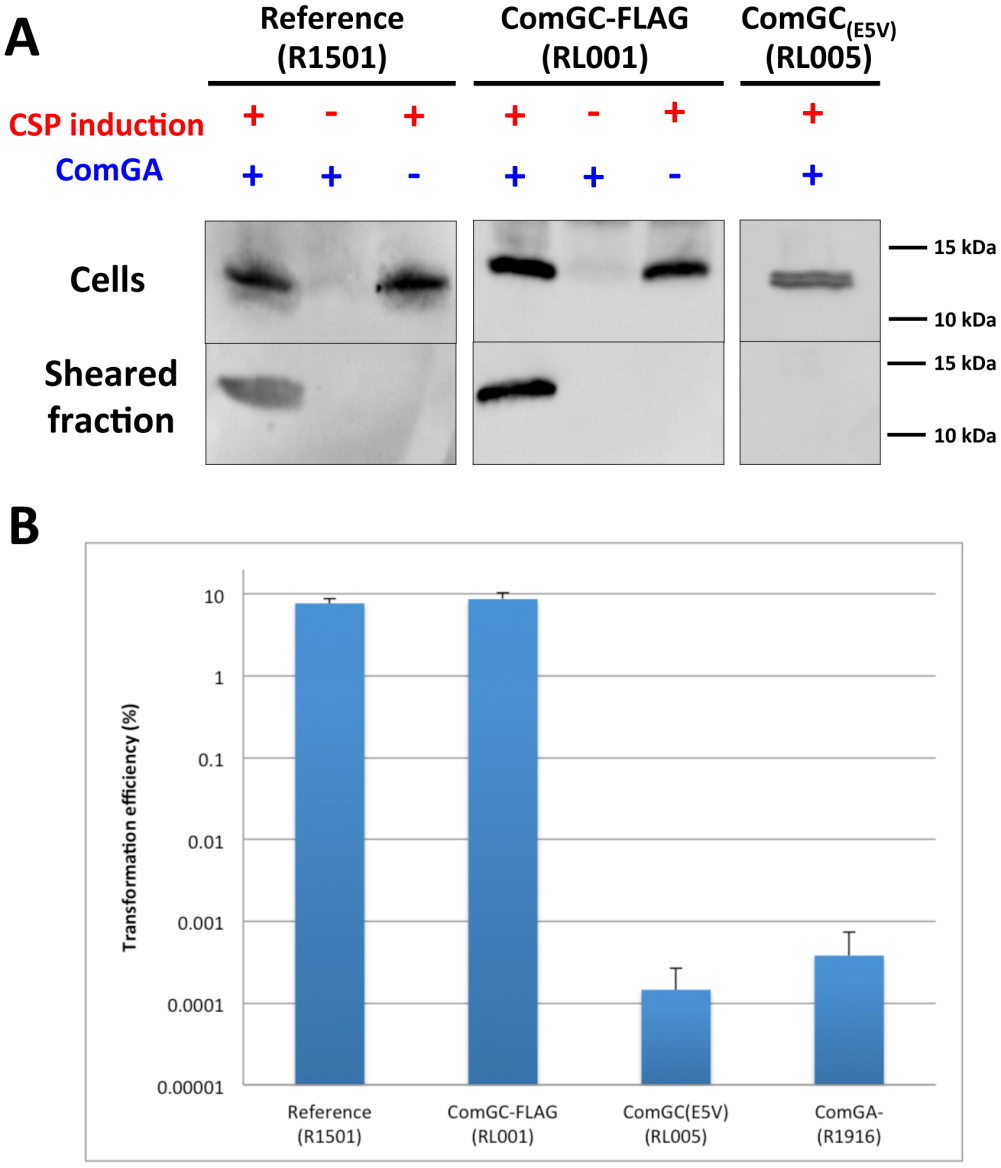
Competence-induced appendages assembly and transformation efficiency. (**A**) Detection of ComGC in the sheared and cellular fractions by immunoblot. ComGC was detected in the sheared fraction of reference (R1501) and ComGC-FLAG (RL001) competent strains. ComGC was not detected in the sheared fraction of cultures that were not competence-induced. Deletion of *comGA* completely abolished detection of the ComGC pilin in the sheared fraction in both reference and ComGC-FLAG strains. Substitution of the conserved glutamic acid in position 5 of the mature ComGC pilin (Figure 1) by alanine (ComGC_(E5V)_) also completely abolished detection of ComGC in the sheared fraction. For the reference and ComGC_(E5V)_ strains, detection of ComGC was performed with a polyclonal rabbit antibody raised against the soluble domain of ComGC. With the ComGC-FLAG strain, the FLAG-tagged pilin was detected by an anti-FLAG monoclonal antibody. (**B**) Transformation assay using the reference strain (R1501), comGC-FLAG strain (RL001), *comGC*_(E5V)_ mutant (RL005) and *comGA* deletion mutant (R1916). Both *comGC*_(E5V)_ and *comGA* mutants were defective for transformation.

Mechanical shearing is frequently used to release bacterial surface appendages and to study their protein composition [16]. To see if ComGC was part of a macromolecular complex at the surface of competent *S. pneumoniae*, we adapted the method to this bacterium and raised antibodies against the purified soluble domain of ComGC. Using this antibody, we showed that ComGC could be detected by immunoblotting in the sheared fraction of competent bacteria (Figure 2A). While ComGC level in the cell fraction was not affected, no ComGC could be found in the sheared fraction in a *comGA* knockout mutant (Figure 2A). These data strongly suggest that ComGC is part of an extra-cellular appendage and that ComGA is necessary to its assembly.

### A ComGC-containing appendage is visualized at the surface of competent pneumococci

**Figure 3:**
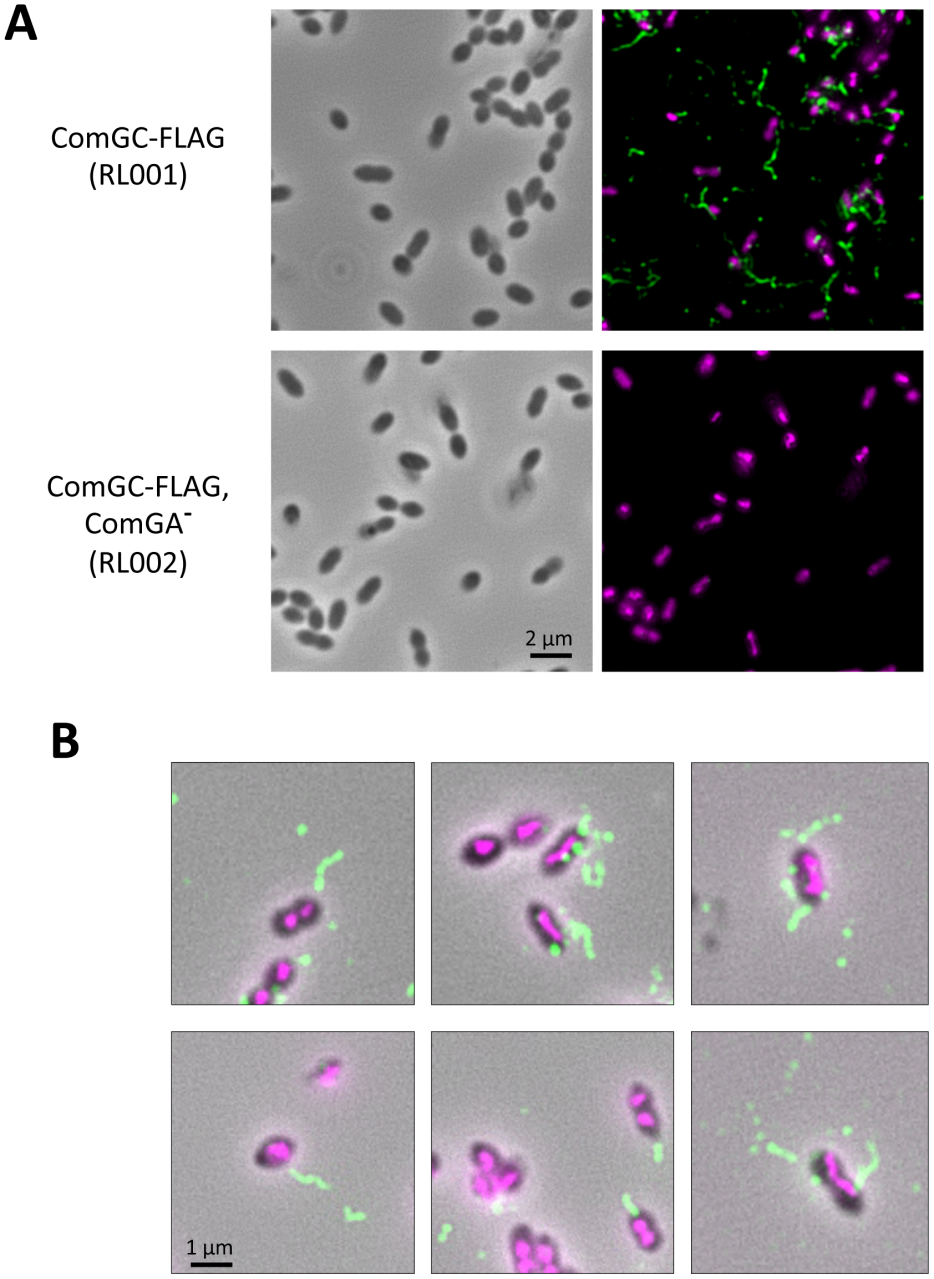
Direct visualization of competence-induced appendages by Immuno-fluorescence. (**A**) Immuno-fluorescence microscopy showing intact competent cells expressing a FLAG-tagged ComGC pilin in presence (top row) or absence of ComGA (bottom row). Left column correspond to bright field image, right column to overlay between anti-FLAG antibody fluorescence (green) and DAPI fluorescence (magenta). (**B**) Zoom on several bacterial cells visualized by immuno-fluorescence. Overlay of bright filed image, anti-FLAG antibody fluorescence (green) and DAPI fluorescence (magenta). Distinct appendages are visible on competent cells.

We inserted a FLAG tag at the C-terminus of ComGC to directly visualize the competence-induced appendages by immuno-fluorescence. It was not possible to insert the sequence encoding the tag at the *comGC* locus on the chromosome because *comGC* and *comGD* genes overlap in the *comG* operon. Therefore, a copy of *comGC* encoding a C-terminally FLAG-tagged ComGC (ComGC-FLAG) was integrated ectopically into the chromosome of *S. pneumoniae* under the control of a competence-induced promoter [17]. The transformation efficiency was not affected in this strain (Figure 2B). Using anti-FLAG antibodies, we could show by immuno-fluorescence that almost all the cells appeared to harbour one or a few ComGC foci or distinct fluorescent appendages (Figure 3A and B; Figure S1A). Due to sample preparation, many broken appendages were also found in the background. No preferential location of the foci/appendages at the cell surface was observed. They are absent in *comGA* knockout cells (Figure 3A). Note that anti-ComGC antibodies were not able to label the competent cells. They probably recognize epitopes that are masked when ComGC is included in the appendages.

### Competence-induced appendage morphology and composition

**Figure 4:**
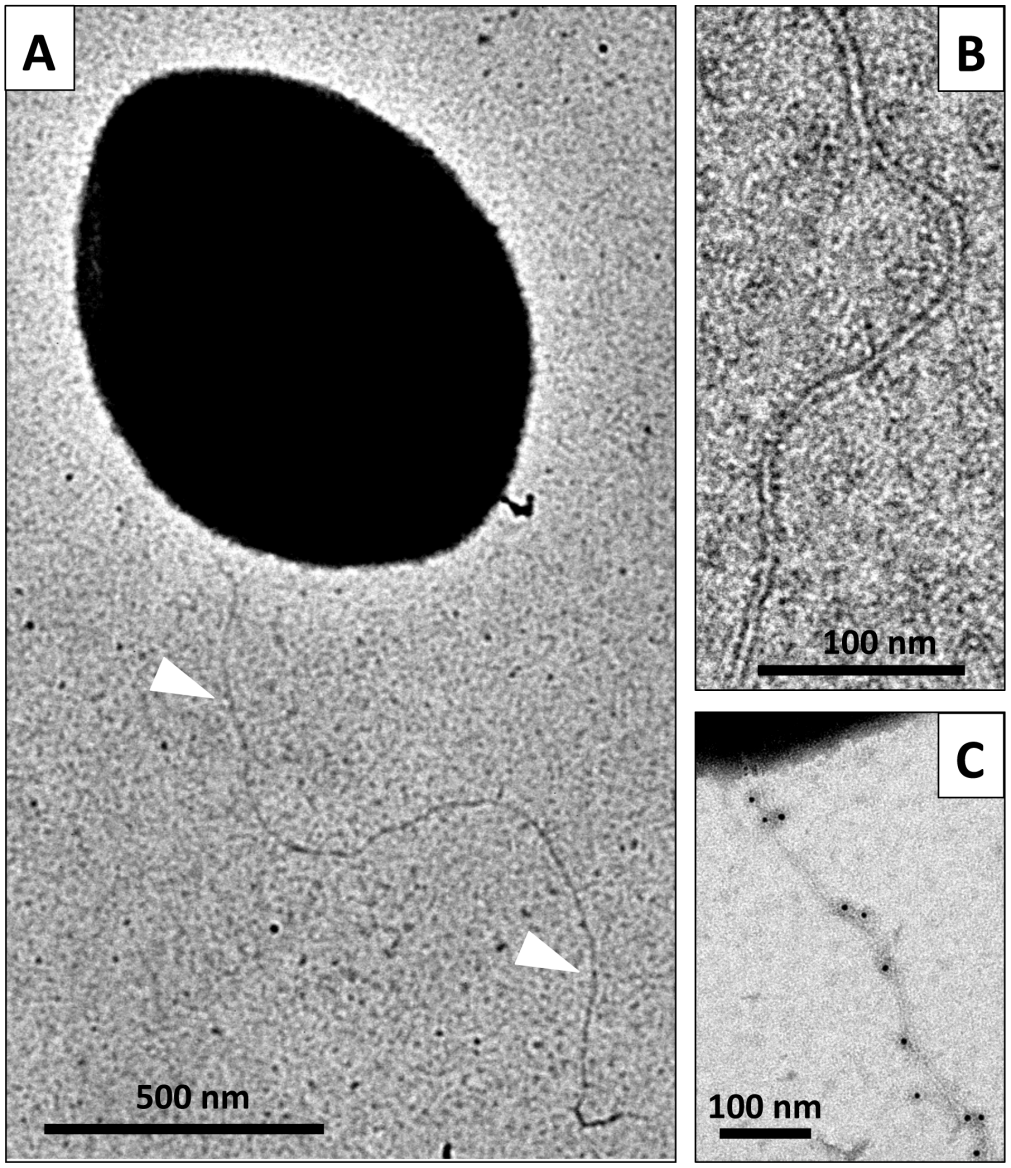
Direct visualization of the competence-induced appendage. Competent reference bacteria observed by transmission electron microscopy. Single appendages, 5–6 nm wide, were observed at the surface most of the competent cells in the culture. Many long filaments were observed, reaching up to several micrometers in length. (**A**) a competent *S. pneumoniae* cell with a long pilus (white triangle). (**B**) closer view of a transformation pilus. (**C**) A pilus observed by transmission electron microscopy after immunogold labeling with anti-FLAG antibody (5 nm gold beads) using the ComGC-FLAG strain. ComGC-FLAG proteins are detected within the appendages.

Using electron microscopy, we observed filaments attached to the cell surface of negatively stained competent pneumococci (Figure 4A and B). These flexible filaments are 5–6 nm in diameter. Their length could reach up to 2–3 micrometers (Figure 4A). A maximum of 2–3 filaments per cell could be observed. Their average length was difficult to assess because they break easily into smaller fragments during sample preparation. Using the ComGC-FLAG expressing strain, we confirmed by immunogold-labelling that they contain ComGC (Figure 4C).

**Figure 5:**
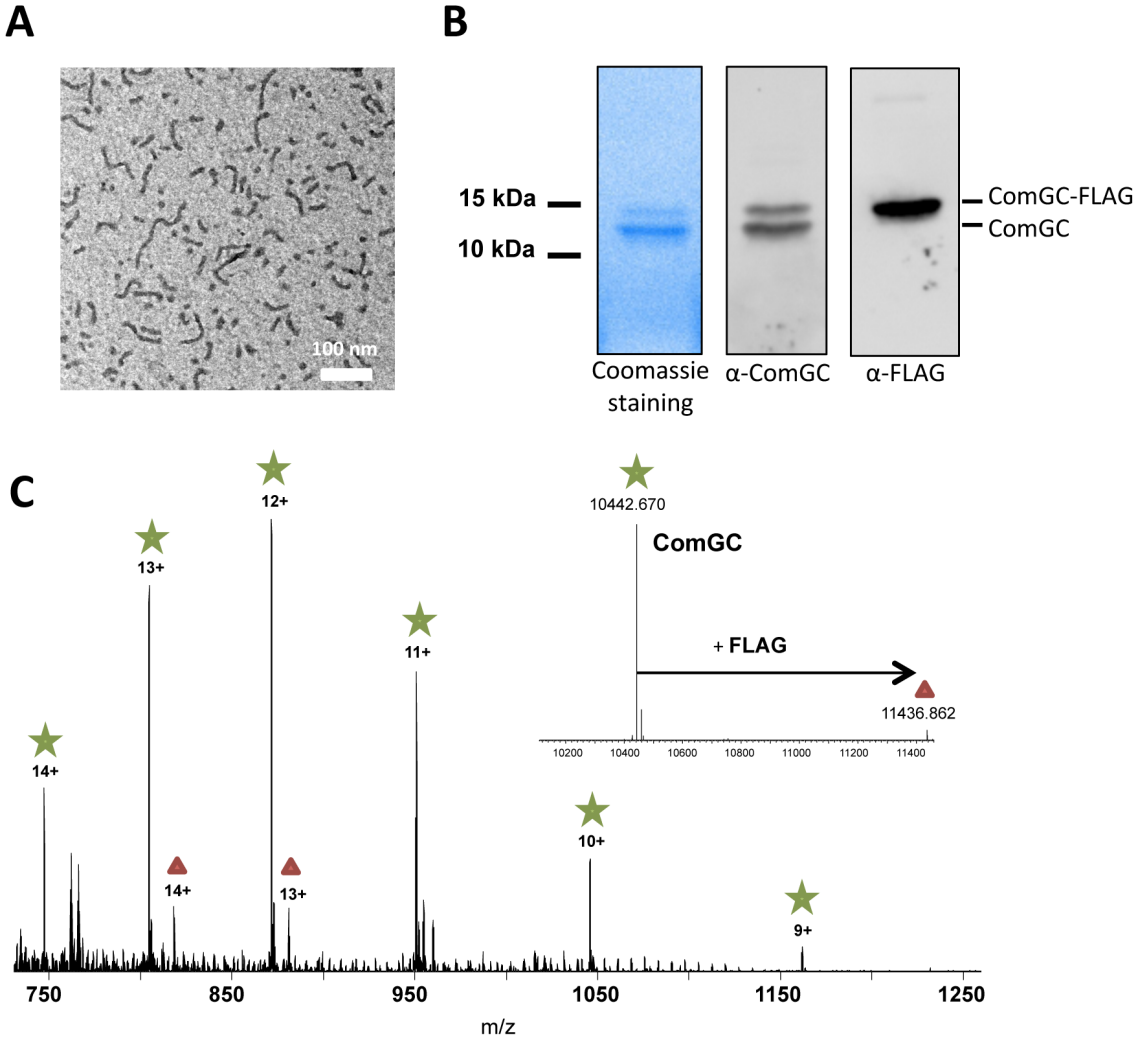
Nature of the transformation pilus. (**A**) Purified pili visualized by negative stain electron microscopy. Short pilus fragments ranging from 50 to 500 nm were observed. (**B**) SDS-PAGE analysis of the purified fraction. Left lane, Coommassie blue staining. Middle lane, Western blot using anti-ComGC antibody. Right lane, Western blot analysis using anti-FLAG analysis. (**C**) Nano-ESI FT-MS spectrum of purified pili. Peaks corresponding to ComGC are labelled with a green star those corresponding to ComGC-FLAG with a red triangle. ComGC-FLAG is present at approximately 6+/−2% abundance of the native form. The deconvoluted spectrum showing monoisotopic masses of the neutral protein forms is presented in the inset. The measured mass of methylated comGC (10,442.670 Da) compares very well to the calculated theoretical mass (10,442.636 Da) with an error of +3 ppm.

Appendages were then purified using anti-FLAG affinity chromatography after mechanical shearing. Appendage fragments of between 50 and 500 nm in length were observed by electron microscopy (Figure 5A), showing that these filamentous structures do not disassemble during purification. SDS-PAGE analysis of the purified fraction showed that ComGC is the major component of the appendages (Figure 5B). Using whole protein mass profiling by high-resolution mass spectrometry [18], we could only detect ComGC and ComGC-FLAG in the purified material (Figure 5C), confirming that ComGC is the major constituent of these appendages. Monoisotopic mass measurements of intact proteins and top-down fragmentation using a variety of activation techniques confirmed that the ComGC prepilin is cleaved after the alanine residue in position 15 and that the first amino acid of the mature protein is methylated, presumably by PilD (Figure S2). Indeed, PilD homologs in Gram-negative bacteria catalyze this post-translational modification of the Type IV pilins [19]. No other post-translational modification was detected in ComGC. Other proteins, including other ComG proteins, were not detected in the purified material by the methods used in this study. This suggests that these proteins are either absent, present in very low amount within the appendage or weakly bound to it and lost during sample preparation. These morphological and biochemical features are typical of Gram-negative Type IV pili. Therefore, we propose that the competence-induced appendage observed in *S. pneumoniae* belongs to the Type IV pilus family.

### The competence-induced pili are required for transformation

It was important to determine whether these competence-induced pili were involved in transformation. Indeed, it was previously shown that *S. pneumoniae* and *B. subtilis comGA* knockout could not be transformed (Figure 2B) [9] [13]. In this study, we were able to show in *S. pneumoniae* that *comGA* mutant cells lack pili (Figure 2A and 3A). It was enticing to conclude that competence-induced pili assembly is essential for transformation. However, it was recently shown that a *comGA* mutation could have a pleiotropic effect on transformation in *B. subtilis* [14]. Therefore, we generated a *comGC* mutant in *S. pneumoniae* in which the conserved glutamic acid in position 5 was substituted by an alanine (Figure 1B). Such a substitution was shown to impair Type IV pilus assembly in Gram-negative bacteria [20]. ComGC cellular level was not affected by this point mutation (Figure 2A). Our results show that this mutant strain could not assemble any pilus and that it was defective for transformation (Figure 2A and B). Therefore we conclude from the analysis of both *comGA* and *comGC_(E5A)_* mutants that the assembly of the competence-induced pilus is required for transformation.

### Transformation pili bind extracellular DNA

**Figure 6:**
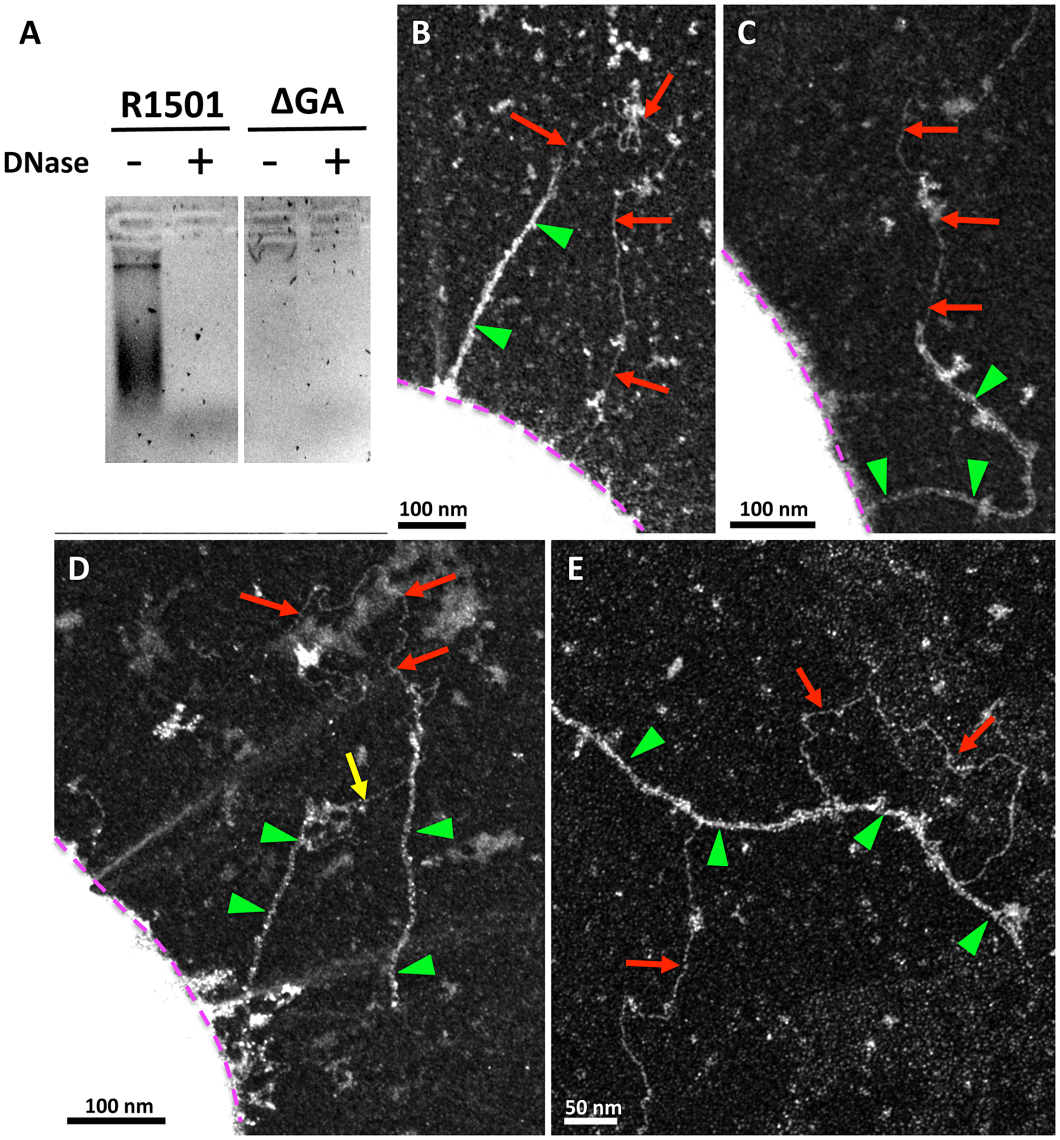
Interactions between transformation pili and DNA. (**A**) DNA co-purified with the pili by affinity chromatography was revealed after migration on agarose gel and Gel Green staining. DNA was present in the ComGC-FLAG strain. In the absence of ComGA, DNA was not detected. DNase treatment unambiguously showed that the bands detected on the gel are DNA. Linear DNA (red arrow) bound to the pilus (green triangle) in *ΔcomFA* (**B**) and *ΔcomEC* (**C**), (**D**) and (**E**) strains. (**B**) and (**C**), linear DNA interacting with a single pilus. The DNA molecule contacts the pilus at several points. **D**, a DNA molecule maintains a broken pilus close to the bacterium (yellow arrow), demonstrating the existence of several DNA binding sites on the pilus. (**E**) Detached pilus found in the medium with bound DNA. In (**B**), (**C**) and (**D**), bacterial cell envelope is highlighted by a purple dotted line.

The nature of the primary DNA receptor at the surface of transformable Gram-positive bacteria is not known. It is generally proposed that the transformation pseudopilus would bind extracellular DNA at the surface of competent Gram-positive bacteria [8], [21]. However, this hypothesis has never been confirmed experimentally. Using affinity purification, we show that DNA naturally released in the culture medium co-fractionates with the purified pili. No DNA could be found in the purified fraction in absence of the pilus (Figure 6A). These data were a first hint suggesting that DNA present in the environment could bind to the transformation pilus. However, it was not clear if this binding was related to the transformation process or fortuitous. By using specific electron microscopy methods [22], we visualized DNA directly bound to the transformation appendage after adding linear double stranded DNA (dsDNA) to competent bacteria. Long stretches of dsDNA interacting with the transformation pilus were observed with clearly visible multiple contact points (Figure 6 B–E). Interestingly, it was extremely difficult to see DNA bound on the pilus in the reference bacteria (R1501 strain), which are known to internalize exogenous DNA quickly [23]. On the other hand, in *ComEC* and *comFA* mutants, we could easily observe bound DNA on transformation pili. These strains are defective for DNA uptake and accumulate bound DNA at their surface [13]. Given that the dsDNA was added in large excess, no difference between the reference and mutant strains should be observed if DNA binding on the pili was a coincidental event. The fact that the uncoupling of DNA binding and uptake processes facilitates the observation of the DNA/pilus interaction is a strong indication that DNA binding on the transformation pilus is related to the transformation process.

## Discussion

The *comG* operon is conserved in all transformable Gram-positive bacteria. This operon encodes proteins that are homologous to proteins involved in Type IV pilus assembly in Gram-negative bacteria. Therefore it has been proposed that a pilus (or pseudopilus) could be assembled at the surface of competent Gram-positive bacteria. Since all *comG* genes are essential for transformation, this pilus could be directly involved in transformation. The first biochemical clues for the existence of a transformation pilus were found in *B. subtilis* although decisive observational support was lacking. In addition, it was not clear if the ComGC-containing macromolecular complex found in *B. subtilis* was a common feature of competent Gram-positive bacteria or specific to this species. Finally, the function of this putative transformation pilus, and in general of the ComG proteins, was unclear.

### Discovery of a new pneumococcal appendage

The pneumococcal transformation pilus represents a newly discovered pneumococcal surface structure. For a long time, no external appendage could be found at the surface of *S. pneumoniae* cells while many electron microscopy images were published in the literature. Recently, sortase-mediated pili have been discovered in some pathogenic *S. pneumoniae* strains [24]. To our knowledge, no specific ultrastructural study of competent *S. pneumoniae* has ever been described. Here, we analysed a laboratory strain that is commonly used to study the transformation process in *S. pneumoniae* [10] [13]. In this strain, competence can be induced in a rapid and synchronous manner upon addition of synthetic CSP in the medium of an exponentially growing culture [5], [25]. To make sure that the appearance of the transformation pilus is a common feature of competent pneumococci and not a mere one-off property of our reference strain, we observed negatively stained G54 and CP strains by electron microscopy. The G54 strain is a wild-type clinical strain. The CP strain is a laboratory strain that has a different genetic background than our reference strain [26]. In both cases, transformation pili were observed at the surface of competent cells (Figure S3). Therefore, we think that transformation pili are found at the surface of most, if not all, pneumoccocal strains, including clinical strains.

### The transformation pilus is a Type IV pilus

The pneumococcal transformation pilus is morphologically very similar to Type IV pili found in many Gram-negative bacteria. Its major component, the ComGC pilin, is cleaved and probably methylated by a PilD homolog. We therefore propose that the transformation pilus is a *bona fide* Type IV pilus. Since its length can reach up to 2–3 µm, we think that the “pseudo-pilus” appellation does not apply to the pneumococcal transformation appendage. By comparison, the type II secretion pseudo-pilus is just 50–100 nm long [27]. The transformation pilus is the first Type IV pilus clearly observed in a Gram-positive bacterium. So far, Type IV pilus-dependent gliding motility had been described in *Clostridium* species [28]. However, no clear picture of this pilus was provided. A recent genomic study show the existence of numerous and diverse Type IV pilus-like operons in a wide range of Gram-positive bacteria [29]. This suggests that many other Type IV-like pili remain to be discovered in these bacteria. The conservation of *comG* operons argues in favor of the presence of a transformation pilus in all naturally transformable Gram-positive bacteria. However, species-specific variations in pilus length can be anticipated because of variations in thickness of the capsule and/or the cell wall.

#### Pilus function

We envision the transformation pilus to act as a “DNA-trap” to capture DNA in the environment. In Gram-negative bacteria, Type IV pilus assembly is also essential for natural transformation [30]. It is proposed that this pilus interacts directly with DNA but the molecular details of this interaction remains enigmatic [31], [32], [33]. No DNA binding protein could be identified in the pilus [34]. Recently a minor pilin, ComP, has been identified as a DNA receptor specific of genus-specific DNA uptake sequence (DUS) motifs in *Neisseria meningitidis* [35]. Our data explicitly show that the pneumoccocal transformation pilus binds DNA. At this stage, it is not clear if this DNA binding ability is due to the physicochemical properties of the pilus and/or due to a yet undetected minor pilin. Interestingly, only ComGA homolog was found indispensable for initial DNA binding at the surface of *B. subtilis* [14]. These bacteria could assemble only short transformation pili that are not sufficient to bind DNA at the surface of the competent cells. An unknown DNA receptor that interacts with ComGA ATPase could be required for efficient DNA binding in this species. It is possible that the mechanism of initial DNA binding at the surface of competent Gram-positive bacteria vary.

#### Pilus assembly and retraction

In Gram-negative bacteria, 12 to 15 proteins are necessary for Type IV pilus biogenesis [36], [37]. The assembly of the pneumococcal transformation pilus could require the simplest Type IV pilus assembly apparatus discovered so far in bacteria. Indeed, only 7 ComG proteins might be sufficient to assemble this pilus. Strikingly, Type IV pili are retractile appendages in Gram-negative bacteria [38]. It is therefore appealing to propose that the transformation pilus could retract to guide bound DNA through the cell wall and the polysaccharide capsule that is often present in bacteria (as in *S. pneumoniae*), and, ultimately deliver it to the ComEA/EndA complex located in the membrane [7]. It is unlikely that this DNA would find its way along several hundreds of nanometers through the cell wall and capsule without pilus retraction. However, a dedicated ATPase called PilT powers pilus retraction in Gram-negative bacteria and *S. pneumoniae* genome does not encode any PilT homolog. All the *comG* operons that have been identified so far in Gram-positive bacteria encode only one ATPase, ComGA, that is required for the pilus assembly. Therefore, the possible retraction of the transformation pilus and role of ComGA in this process should be assessed.

Our data clearly establish the existence and function of a transformation pilus at the surface of competent pneumococci. As an essential component of the transformation apparatus, it enables this major human Gram-positive pathogen to acquire resistance to antibiotics and to escape vaccines through the binding and incorporation of new genetic material. Future work should establish whether transformation pili exist and play similar roles in other transformable pathogens. Intriguingly, ComG proteins also appear to play an important and direct role in phagosomal escape and virulence in *Listeria monocytogenes* [39]. It would be interesting to determine whether they also assemble into a pilus to play this role.

## Materials and Methods

### Strain construction and growth

Cells were grown at 37°C under anaerobic condition, without agitation, in a Casamino Acid Tryptone medium (CAT) up to OD_600_ = 0.3 for stock cultures [40]. After addition of 15% glycerol, stocks were kept frozen at −80°C. For competence induction, cells were grown in CAT supplemented with BSA (2 g/L), calcium chloride (1 mM) and adjusted to pH = 7.8. Competence was triggered by incubating cells with the Competence Stimulating Peptide (CSP) at OD_600_ = 0.1 for 12 min as described previously [40]. For transformation, DNA was then added and transformants were selected on CAT agar plates [17]. Competence was induced following the same protocol in G54 and TCP1251 strains.

For transformation efficiency assays, 100 µL of competent bacteria were transformed by the addition of 100 ng of *S. pneumoniae* R304 genomic DNA (contains the streptomycin resistance gene *str41*). Bacteria were plated in presence and absence of streptomycin (100 µg/mL final concentration) and incubated at 37°C overnight before colony counting.

The annotated names of the *comG* genes in different strains of *S. pneumoniae* are listed in Table S1. The *S. pneumoniae* strains used derived from the non-capsulated R6 strain and are listed in Table S2. The *comGC-FLAG* gene was cloned by PCR using genomic DNA of pneumococcal R6 strain (ATCC BAA-255) as template. The resulting fragment was digested with *NcoI* and *BamHI* and inserted into the same sites of the pCEPx vector [17]. RL001 strain was constructed by transformation of R1501 cells with the pCEPx plasmid containing *comGC-FLAG*, followed by selection with kanamycin (Kan). RL002 was obtained by transformation of RL001 with R1062 chromosomal DNA and selection with spectinomycin (Spc). For RL003, a 2 kb genomic fragment of R6 genome containing *comGC* in the middle was amplified, and the codon 20 was changed from GAG to GTG by cross-over PCR. R1501 was transformed with this modified genomic fragment, and clones were screened by sequencing the *comGC* gene.

Chemically competent *Escherichia coli* BL21 Star (Life Technologies) were used for heterologous production of ComGC soluble domain. The corresponding DNA sequence was amplified from genomic DNA of strain R800 and cloned into pET15b expression vector (Novagen), using *NdeI/XhoI*. The protein was purified from the soluble fraction using IMAC affinity and gel filtration in 50 mM Tris/HCl pH = 8, 200 mM NaCl. The anti-ComGC were raised against the purified protein (Eurogentec).

### Detection and purification of cell surface appendages

Shearing experiments were adapted from Sauvonnet et al. [41]. Competence was induced exactly as described above in a 50 mL culture. Cells were harvested by centrifugation 15 min at 4,500 g, 4°C. The pellet was suspended in 1 mL LB and immediately vortexed for 1 min to apply mechanical pressure. The suspension was then centrifuged twice at 13,000 g for 5 min to separate the bacteria (pellet fraction) from the pilus-enriched supernatant (sheared fraction). The supernatant was then precipitated with 10% trichloroacetic acid for 30 min on ice. Both fractions were loaded on SDS 15% polyacrylamide gels and subjected to electrophoresis and immunoblot with rabbit polyclonal antibodies raised against ComGC soluble domain (38–108) or anti-FLAG M2 antibody (Sigma-Aldrich F1804).

The pili containing ComGC-FLAG were purified from the sheared fraction of a 1 L culture. Shearing was performed in 2 mL Tris Buffered Saline (TBS, Tris pH 7.6 0.05 M, NaCl 0.15 M, protease inhibitor cocktail Roche 11873580001) and incubated overnight on a rotating wheel at 4°C with ANTI-FLAG M2 affinity resin (Sigma-Aldrich A2220). After washing with TBS, the pili were eluted by adding 3×FLAG-peptide at 100 µg/mL (Sigma Aldrich F4799) 30 min at room temperature under agitation.

To prevent DNA aspecific binding on the ANTI-FLAG M2 affinity resin, the resin was saturated 2 h at 4°C with a 1.5 kb PCR fragment (20 ng/µL). For DNA detection, 20 µL of the eluted pili were run on a 1% agarose gel and stained with SYBR safe (Life technologies S33102).

### Visualization of pili and immunogold labelling

Competence was induced exactly as described above in a 10 mL culture. Cells were harvested by centrifugation 15 min at 4,500 g, 4°C. The pellet was suspended in 60 µL phosphate-buffered saline (PBS) (Sigma-Aldrich P4417). A drop of this suspension was placed on a glow discharged carbon coated grid (EMS, USA) for 1 min. The grid was then placed on a drop of PBS-3% formaldehyde, 0.2% glutaraldehyde for 10 min, and washed on drops of distilled water. The grids were then treated with 2% uranyl acetate in water. Specimens were examined using a Philips CM12 transmission electron microscope operated at 120 kV. Pictures were recorded using a camera KeenView (SIS, Germany) and ITEM software. For immunogold labelling, additional steps were applied after fixation: 3 washes with PBS, PBS–50 mM NH_4_Cl (10 min), 3 washes with PBS, PBS with 1% BSA (5 min), 1 hour incubation with ANTI-FLAG M2 antibody (Sigma-Aldrich F1804) diluted 1/100 in PBS with 1% BSA, 3 washes with PBS-BSA 1% (5 min), 1 hour incubation with goat anti-mouse antibody (5 nm gold particles, BritishBioCell, UK) diluted 1/25 in PBS containing 1% BSA.

### Fluorescence microscopy

*S. pneumoniae* cells were grown in the same conditions as above for visualization by electron microscopy. Cells were harvested by centrifugation for 15 min at 4,500 g, 4°C. The pellet was suspended in 500 µL PBS and directly immobilized on poly-L-lysine-coated coverslips. Samples were fixed for 30 min with 3.7% formaldehyde, washed 3 times with PBS containing 1% BSA and incubated on a 100 µL drop of anti-FLAG antibodies (1∶300) and secondary Alexa Fluor 488- coupled anti-mouse IgG (Invitrogen). Samples were examined with an Axio Imager.A2 microscope (Zeiss). Images were taken with AxioVision (Zeiss) and processed in ImageJ [42].

### Mass spectrometry

Protein samples were desalted and eluted directly into a 10 µL spray solution of methanol∶water∶formic acid (75∶25∶3). Approximately 4 µL was loaded into a coated, medium sized, nano-ESI capillary (Proxeon) and introduced into an Orbitrap Velos mass spectrometer, equipped with ETD module (Thermo Fisher Scientific, Bremen, Germany) using the off-line nanospray source in positive ion mode. A full set of automated positive ion calibrations was performed immediately prior to mass measurement. The transfer capillary temperature was lowered to 100°C, sheath and axillary gasses switched off and source transfer parameters optimised using the auto tune feature. Helium was used as the collision gas in the linear ion trap. For MSn experiments, ions were selected with a 3 Da window and both CID and HCD were performed at normalised collision energies of 15–25%, with the appropriate HCD charge state set and other activation parameters left as default. For ETD the reagent gas was fluoranthene and the interaction time 10 ms. Supplemental activation was used as noted. The FT automatic gain control (AGC) was set at 1×10^6^ for MS and 2×10^5^ for MSn experiments. Spectra were acquired in the FTMS over several minutes with one microscan and a resolution of 60,000 @ m/z 400 before being summed using Qualbrowser in Thermo Xcalibur 2.1. Summed spectra were then deconvoluted using Xtract and a, b, c−1, y, z, z+1 ions assigned using in house software at a tolerance of 5 ppm. N-terminal ions were verified manually.

### Positive staining electron microscopy

Five microliters of bacterial culture (wild-type, *ΔcomFA* or *ΔcomEC*) were diluted in 45 µL of Tris 10 mM, pH 8, NaCl 150 mM. Bacteriophage lambda DNA (0,1 mg/ml final) was then added to bacteria. Five µL of mix were immediately adsorbed onto a 600 mesh copper grid coated with a thin carbon film, activated by glow-discharge. After 1 min, grids were washed with 0,02% (w/vol) uranyl acetate solution (Merck, France) and then dried with filter paper. TEM observations were carried out with a Zeiss 912AB transmission electron microscope in filtered crystallographic dark field mode. Electron micrographs were obtained using a ProScan 1024 HSC digital camera and Soft Imaging Software system.

## Supporting Information

Figure S1 **Visualization of competence-induced appendages by Immuno-fluorescence.** Same picture as in figure 3A, in high resolution. Left column correspond to bright field image, right column to overlay between anti-FLAG antibody fluorescence (green) and DAPI fluorescence (magenta).
(PDF)

Figure S2 **Mass spectrometry analysis of the major pilus component.** Fragment map of GomGC generated from several top-down mass spectrometry experiments. Sequence coverage is 74%. MS/MS spectra formed through different fragmentation techniques were deconvoluted and de-isotoped in Xtract and the resulting peak lists combined. Fragment peaks were picked and assigned from this combined list using in house software at a tolerance of 5 ppm. Individual experimental conditions were as follows; ETD 14+ charge state 7 ms activation time; 13+ charge state 10 ms activation time, 5 ms activation time with and without supplementary activation; HCD 30 eV collision energy, 13 eV collision energy; CAD 20 eV collision energy.
(PDF)

Figure S3 **Transformation pili are observed in other pneumococcal strains.** Competent G54 and TCP1251 *S. pneumoniae* cells were observed by transmission electron microscopy. The same appendages were detected in these strains.
(PDF)

Table S1 ***ComG*** **and** ***pilD*** **genes in different pneumococcal strains.** The name used to designate the comG and pilD genes varies in different pneumoccocal strains. For clarity, we refer to the comG nomenclature used in B. subtilis. Names of the corresponding genes in different S. pneumoniae strains are found in the table.
(DOCX)

Table S2 **Strains and plasmids.** The strains and plasmids used in this study are listed in this table.
(DOCX)
