# Supplementary figures and images for: A Type IV Pilus Mediates DNA Binding during Natural Transformation in Streptococcus pneumoniae
Source: PLoS Pathog. 2013 Jun 27;9(6):e1003473. doi: 10.1371/journal.ppat.1003473 (PMC3694846; doi:10.1371/journal.ppat.1003473)

ComGC-FLAG  
(RL001)

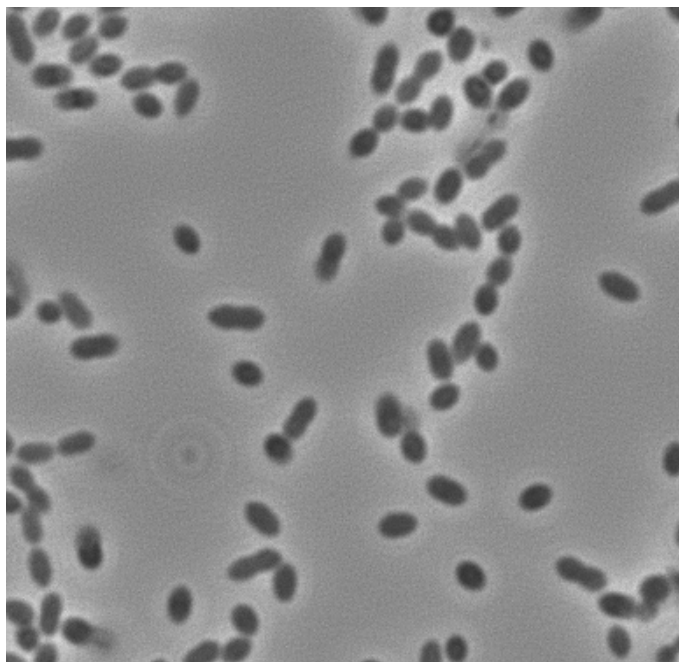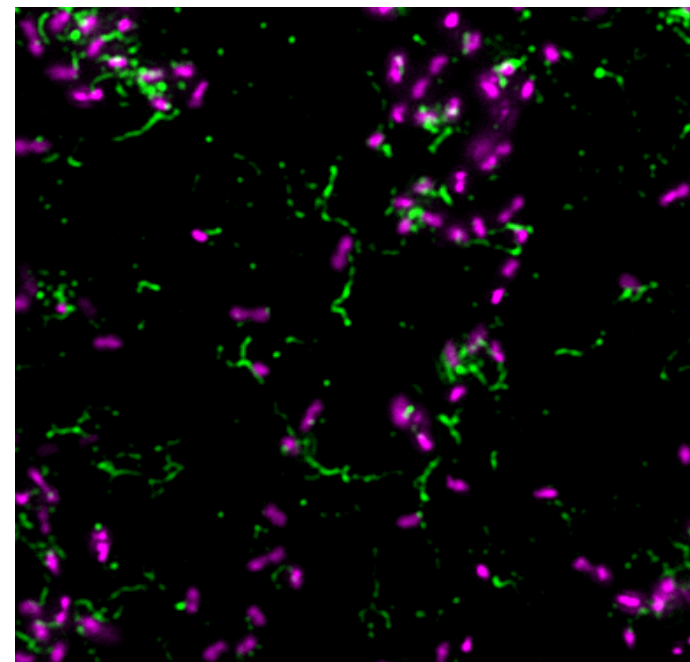

ComGC-FLAG, ComGA<sup>-</sup>  
(RL002)

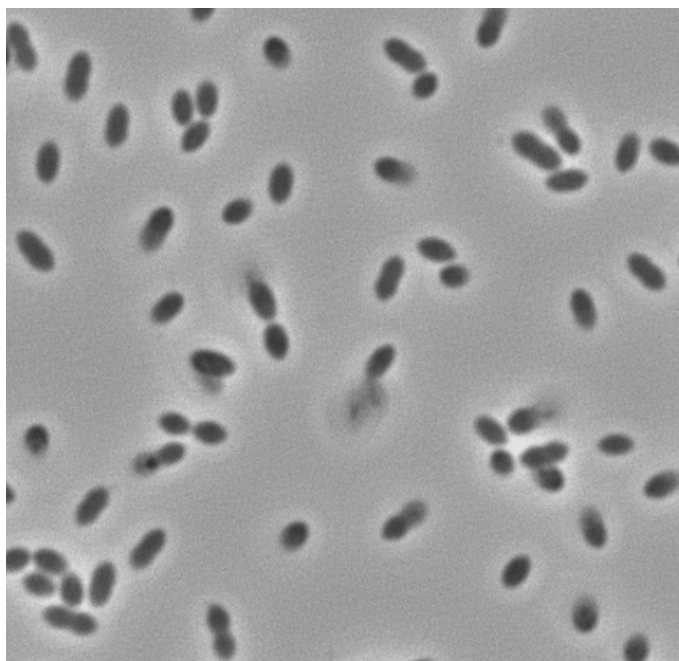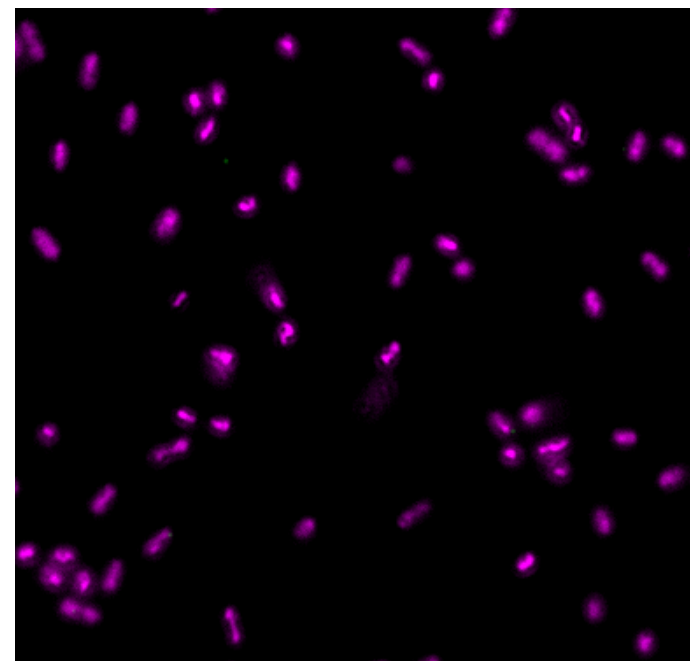

Supplement: Figure S1 — Visualization of competence-induced appendages by Immuno-fluorescence. Same picture as in figure 3A, in high resolution. Left column correspond to bright field image, right column to overlay between anti-FLAG antibody fluorescence (green) and DAPI fluorescence (magenta). (PDF) [file ppat.1003473.s001.pdf]

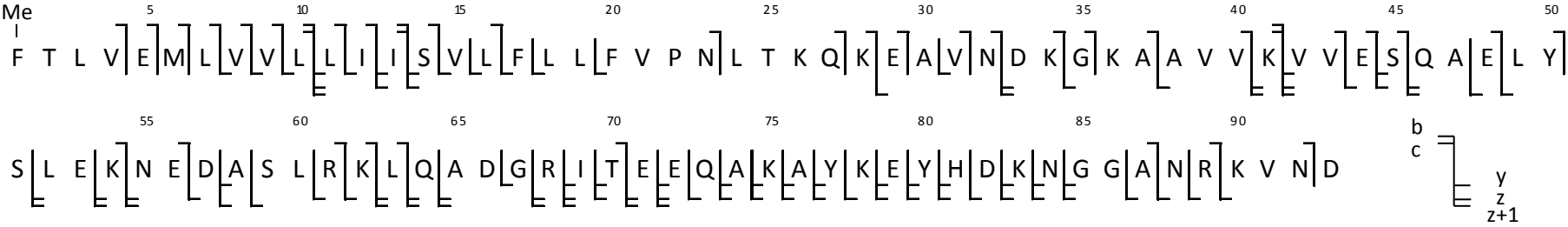

Supplement: Figure S2 — Mass spectrometry analysis of the major pilus component. Fragment map of GomGC generated from several top-down mass spectrometry experiments. Sequence coverage is 74%. MS/MS spectra formed through different fragmentation techniques were deconvoluted and de-isotoped in Xtract and the resulting peak lists combined. Fragment peaks were picked and assigned from this combined list using in house software at a tolerance of 5 ppm. Individual experimental conditions were as follows; ETD 14+ charge state 7 ms activation time; 13+ charge state 10 ms activation time, 5 ms activation time with and without supplementary activation; HCD 30 eV collision energy, 13 eV collision energy; CAD 20 eV collision energy. (PDF) [file ppat.1003473.s002.pdf]

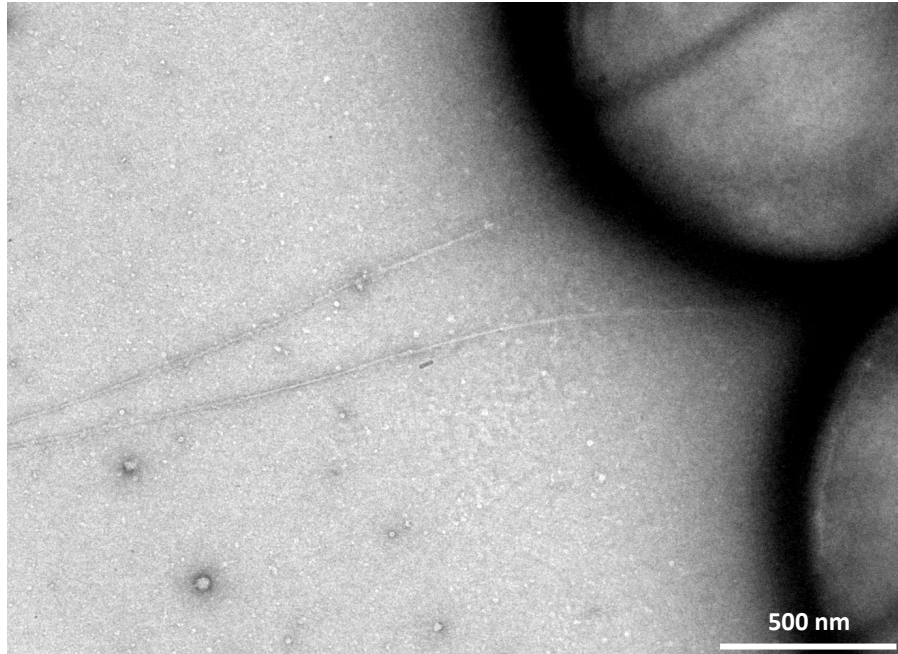

*S. Pneumoniae* G54 strain  
(capsulated)

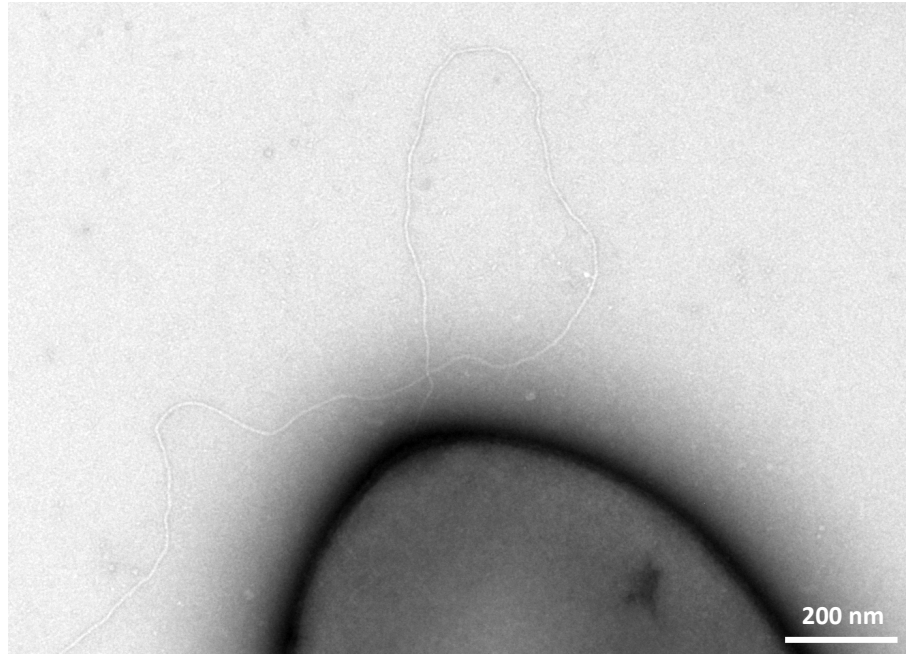

*S. Pneumoniae* TCP1251 strain

Supplement: Figure S3 — Transformation pili are observed in other pneumococcal strains. Competent G54 and TCP1251 S. pneumoniae cells were observed by transmission electron microscopy. The same appendages were detected in these strains. (PDF) [file ppat.1003473.s003.pdf]
