# Supplementary material for: A Type IV Pilus Mediates DNA Binding during Natural Transformation in Streptococcus pneumoniae
Source: PLoS Pathog. 2013 Jun 27;9(6):e1003473. doi: 10.1371/journal.ppat.1003473 (PMC3694846; doi:10.1371/journal.ppat.1003473)
Supplement: Table S1 — ComG and pilD genes in different pneumococcal strains. The name used to designate the comG and pilD genes varies in different pneumoccocal strains. For clarity, we refer to the comG nomenclature used in B. subtilis. Names of the corresponding genes in different S. pneumoniae strains are found in the table. (DOCX) [file ppat.1003473.s004.docx]

**Table S1**

| Strain | *comGA* | *comGB* | *comGC* | *comGD* | *comGE* | *comGF* | *comGG* | *pilD* |
| --- | --- | --- | --- | --- | --- | --- | --- | --- |
| R6 | *spr1864*  */cglA* | *spr1863*  */cglB* | *spr_1862*  */cglC* | *spr_1861*  */cglD* | *Not annotated* | *spr_1859* | *spr_1858* | *pilD/spr1628* |
| D39 | *SPD_1863*  */cglA* | *SPD_1862*  */cglB* | *SPD_1861*  */cglC* | *SPD_1860*  */cglD* | *SPD_1859* | *SPD_1858* | *SPD_1857* | *SPD_1593* |
| G54 | *SPG_1968* | *SPG_1967* | *SPG_1966* | *SPG_1965* | *SPG_1964* | *SPG_1963* | *SPG_1962* | *SPG_1704* |
| CP | *Not Sequenced* | *Not Sequenced* | *Not Sequenced* | *Not Sequenced* | *Not Sequenced* | *Not Sequenced* | *Not Sequenced* | *Not Sequenced* |
| TIGR4 | *SP_2053* | *SP_2052* | *SP_2051* | *SP_2050* | *SP_2049* | *SP_2048* | *SP_2047* | *SP_1808* |
