# Supplementary material for: A Type IV Pilus Mediates DNA Binding during Natural Transformation in Streptococcus pneumoniae
Source: PLoS Pathog. 2013 Jun 27;9(6):e1003473. doi: 10.1371/journal.ppat.1003473 (PMC3694846; doi:10.1371/journal.ppat.1003473)
Supplement: Table S2 — Strains and plasmids. The strains and plasmids used in this study are listed in this table. (DOCX) [file ppat.1003473.s005.docx]

**Table S2**

| Strain number | Genotype/relevant feature^a^ | Reference |
| --- | --- | --- |
| R800 | R6 derivative | [[1](#_ENREF_1)] |
| G54 | Clinical isolate of serotype 19F | [[2](#_ENREF_2)] |
| TCP1251 | Rx derivative but *malM511, rpsL1, bgl1; Sm^R^* | [[3](#_ENREF_3)] |
| R1501 | R800 but ∆*comC* | [[4](#_ENREF_4)] |
| R304 | R800 derivative, *nov1, rif23, str41; Nov^R^, Rif^R^, Sm^R^* | [[5](#_ENREF_5)] |
| R1916 | R1501 but *ssbB::luc (ssbB^+^), comGA::kan; Cm^R^, Kan^R^* | Claverys’ strain collection |
| R998 | R1501 but *comEC::spc; Cm^R^, Spc^R^* | Claverys’ strain collection |
| R1063 | R1501 but *comFA::spc; Cm^R^, Spc^R^* | Claverys’ strain collection |
| RL001 | R1501, but CEPx-*comGC-FLAG* (from plasmid pCEPx-*comGC-FLAG*); *Kan^R^* | This study |
| RL002 | RL001 , but *comGA::spc3^C^* (from strain R1062); *Kan^R^, Spc^R^* | This study |
| RL003 | R1501 but *comGC* E20A (point mutation of ComGC pilin) | This study |
|  | | |
| Plasmids |  |  |
| pCEPx | ColE1 (pBR322) derivative containing the ComX-dependent promoter, P_X_, and the RBS of ssbB; *Kan^R^* | [[6](#_ENREF_6)] |

^R^, resistance.

1. Lefevre JC, Claverys JP, Sicard AM (1979) Donor deoxyribonucleic acid length and marker effect in pneumococcal transformation. Journal of bacteriology 138: 80-86.

2. Dopazo J, Mendoza A, Herrero J, Caldara F, Humbert Y, et al. (2001) Annotated draft genomic sequence from a Streptococcus pneumoniae type 19F clinical isolate. Microbial drug resistance 7: 99-125.

3. Pestova EV, Havarstein LS, Morrison DA (1996) Regulation of competence for genetic transformation in Streptococcus pneumoniae by an auto-induced peptide pheromone and a two-component regulatory system. Molecular microbiology 21: 853-862.

4. Dagkessamanskaia A, Moscoso M, Henard V, Guiral S, Overweg K, et al. (2004) Interconnection of competence, stress and CiaR regulons in Streptococcus pneumoniae: competence triggers stationary phase autolysis of ciaR mutant cells. Molecular microbiology 51: 1071-1086.

5. Mortier-Barriere I, de Saizieu A, Claverys JP, Martin B (1998) Competence-specific induction of recA is required for full recombination proficiency during transformation in Streptococcus pneumoniae. Molecular microbiology 27: 159-170.

6. Martin B, Granadel C, Campo N, Henard V, Prudhomme M, et al. (2010) Expression and maintenance of ComD-ComE, the two-component signal-transduction system that controls competence of Streptococcus pneumoniae. Molecular microbiology 75: 1513-1528.
